# Myiase orbitaire: un cas historique

**DOI:** 10.11604/pamj.2014.19.115.4667

**Published:** 2014-10-01

**Authors:** Mohammed El Mellaoui, Abdelkader Laktaoui

**Affiliations:** 1 Service d'Ophtalmologie, Hôpital Militaire Moulay Ismaïl, Meknès, Maroc

**Keywords:** Myiase orbitaire, cavité orbitaire, larves d'œstrus ovis, orbital myiasis, orbital cavity, oestrus ovis larva

## Image en medicine

Un patient âgé de 68 ans, vivant dans une région rurale et ayant subit une exentération gauche suite à un mélanome choroïdien, consulte aux urgences pour la présence de vers dans la cavité orbitaire gauche depuis 03 jours avec sensation de douleur et de démangeaisons. L'examen trouve un grand nombre de vers très mobiles remplissant la cavité orbitaire gauche et la faisant ressembler à un véritable nid d'asticots. Le retrait des vers a été effectué à la pince sous anesthésie locale. Un traitement associant des soins locaux et une antibiothérapie par voie générale a été instauré. L'examen parasitologique a permis d'identifier les larves d'œstrus ovis, petite mouche grise qui pond ses œufs de façon accidentelle sur l'œil de l'homme en particulier en période estivale. L’évolution a été marquée par la disparition complète des vers avec restauration d'une cavité orbitaire propre. Le patient fut adressé pour chirurgie réparatrice.

**Figure 1:**
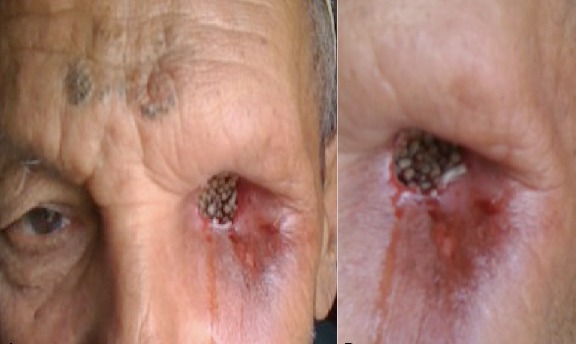
Aspect en nid d'asticots de l'orbite par les larves d'œstrus ovis

